# Pancreatic Fistula after Pancreatectomy: Definitions, Risk Factors, Preventive Measures, and Management—Review

**DOI:** 10.1155/2012/602478

**Published:** 2012-04-24

**Authors:** Norman Oneil Machado

**Affiliations:** Department of Surgery, Sultan Qaboos University Hospital, 123 Muscat, Oman

## Abstract

Resection of pancreas, in particular pancreaticoduodenectomy, is a complex procedure, commonly performed in appropriately selected patients with benign and malignant disease of the pancreas and periampullary region. Despite significant improvements in the safety and efficacy of pancreatic surgery, pancreaticoenteric anastomosis continues to be the “Achilles heel” of pancreaticoduodenectomy, due to its association with a measurable risk of leakage or failure of healing, leading to pancreatic fistula. The morbidity rate after pancreaticoduodenectomy remains high in the range of 30% to 65%, although the mortality has significantly dropped to below 5%. Most of these complications are related to pancreatic fistula, with serious complications of intra-abdominal abscess, postoperative bleeding, and multiorgan failure. Several pharmacological and technical interventions have been suggested to decrease the pancreatic fistula rate, but the results have been controversial. This paper considers definition and classification of pancreatic fistula, risk factors, and preventive approach and offers management strategy when they do occur.

## 1. Introduction

Pancreaticoduodenectomy (PD) is one of the standard treatments for various benign and malignant disease of the pancreatic head and periampullary region and distal pancreatectomy for lesions in the tail of pancreas. Recently the operative mortality after PD has significantly declined to 3 to 5%, while the incidence of postoperative morbidity remains high ranging from 30% to 65% [1–8]. The single most significant cause of morbidity and mortality after PD is the development of pancreatic leak and fistula. The leakage rate according to recent reports varies from 0% to 25% depending on the definition used [6–8]. Abdominal abscess and haemorrhage are common sequelae of pancreatic anastomotic leakage which have often been associated with a mortality rate of 40% or more [1–9]. Pancreatic fistula (PF) hence has been one of the major complications discouraging surgeons from performing PD. Recent literature suggests that many factors influence pancreatic leakage after PD, including sex, age, jaundice, operative time, intraoperative blood loss, pancreaticojejunal anastomotic technique, texture of the remnant pancreas, pancreatic duct size, use of somatostatin, and surgeons experience [1–12]. Various strategies have been employed to decrease the incidence of PF including pharmacological manipulations and refinements and modifications in surgical techniques, which are reviewed here.

## 2. Definitions

There is no universally accepted definition of PF. Most definitions of fistula rely on amylase content from an intra-abdominal drain as well as the daily volume of effluent [2–6] Table 1. Yet in the past decade there has been considerable debate as to what threshold of amylase level defines a PF [2–6]. In a recent study, Bassi et al. examined 26 definitions of PF published between 1991 and 2000 [10]. Each definition was arbitrarily assigned a score based on daily fluid output criteria and the timing of fistula development (i.e., number of days from onset and/or duration of fistula). The results revealed wide variations in the incidence of PF from 10% to 29% depending upon the definition [10]. A special study group, now known as the international study group of PF (ISGPF) involving 37 notable pancreatic surgeons from 15 countries, came up with a definition of PF to facilitate comparison of various studies [8]. The definition was extended to standardizing of postoperative treatment. The essential component of an anastomotic leak was the high amylase content (>3 times the upper normal serum value) at any time on or after 3rd postoperative day. The issue was however further compounded by the concept of “clinical relevance”, a phrase often employed to distinguish asymptomatic biochemical PF from those that are associated with clinical illness, therapeutic intervention, or death [11]. The ISGPF adopted and modified the definition based on clinical impact on the patients hospital course and eventual outcome and graded PF into grade (A, B, C) (Table 2) [3–6]. The grading was based on 9 clinical criteria (patient's condition, use of specific treatment, ultrasound and or CT findings, persistent drainage longer than 3 weeks, reoperation, and death, signs of infection, sepsis, and readmissions [3–6].

However the applicability and utility of ISGPF definition in allowing uniform comparison of fistula rates have been questioned by some workers [12]. Recently Strasberg et al. proposed that intra-abdominal collection along with hemorrhage and peritonitis is also the result of pancreaticoanastomotic failure (PAF) which includes the entire spectrum of clinically relevant problem associated with the loss of integrity of pancreaticoenterostomy [12]. They also sought to categorise fistula that occur after distal pancreatectomy (DP) or segmental resection and enucleation, situations that do not involve pancreaticoenterostomy and hence an entity that is distinct from fistula occurring after PD. These fistulas were termed as pancreatic occlusion failure (POF). POF commonly runs a more benign course compared to PAF, since enzyme activation does not occur in the absence of pancreaticoenteric anastomosis [12].

## 3. Risk Factors for Pancreatic Anastomotic Leakage after PD

Risk factors for pancreatic leakage include general patient-related risk factors (age, gender, jaundice, and malnutrition), disease-related risk factors (pancreatic pathology, pancreatic texture, pancreatic duct size, pancreatic juice output), and procedure related factors (operative time, resection type, anastomotic technique, intraoperative blood loss) [1–9]. In addition, surgeons experience has been shown to correlate with pancreatic anastomotic leakage rate and in some reported cases the prophylactic use of somatostatin [2–6].

## 4. Pancreas and Disease-Related Risk Factors

The most widely recognized risk factors for pancreatic fistula are directly linked to state and disease of the pancreas and or/periampullary region [3–9]. Principal among them is a soft pancreatic parenchyma [3, 6]. In a series of nearly 2000 pancreaticoduodenectomies, it was noted that a soft pancreas was associated with a 22.6% fistula rate and led to a 10-fold increased risk of pancreatic fistula versus an intermediate or hard gland [13]. Other investigations have similarly reported high rates of pancreatic fistula in the presence of soft pancreatic parenchyma [3–6, 14, 15]. In other reports, while 25% of patients with soft pancreatic texture were found to be complicated with pancreatic leak, none of the patients with hard pancreatic remnants developed pancreatic leakage [14, 16]. It has been widely accepted that a fibrotic pancreatic remnant in patients with chronic pancreatitis facilitates pancreaticoenteric anastomosis, whereas a soft and friable pancreatic parenchyma makes the anastomosis difficult to perform [3–6]. Hence a strong association between pancreatic texture and pancreatic leakage is found. 

The size of the pancreatic duct has been implicated as a major predictor of fistula [14]. This is particularly so when small nondilated pancreatic ducts, typically defined as less than or equal to 3 mm in diameter, predispose patients to pancreatic fistulae, compared to 7% of patients with dilated ducts [3, 5, 6]. Other disease-related risk factors include resection of pathologic lesions like ampullary or duodenal carcinoma, distal cholangiocarcinoma, intraductal papillary mucinous neoplasia, pancreatic cystadenomas, benign islet tumours, duodenal adenomas, and increased pancreatic juice output [13, 17].

## 5. Patient-Related Risk Factors

Patient characteristics have also been considered as predictive factors for pancreatic fistula including male sex, advanced age (>70 years), identifiable jaundice, creatinine clearance abnormality, and intraoperative blood loss and coronary artery disease [2–9]. A number of studies, including prospective, have found patient's age greater than 70 years as the only factor associated with poor anastomotic healing leading to pancreatic fistula [13, 16]. A fourfold increased likelihood has been found in patients with coronary artery disease, in multivariate analysis [13]. This is likely to be related to impaired anastomotic healing due to impaired visceral perfusion. Jaundice and creatinine clearance have been previously reported to be patient-related risk factors, predisposing to pancreatic fistula after PD [13]. The duration of jaundice rather than the extent of jaundice is found to influence this poor outcome [18]. Yeh et al. demonstrated that the average duration of jaundice among patients with pancreatic fistula was nearly twice as long as that among patients in no fistula group: 45 ± 21 days versus 23 ± 11 days (*P* = 0.018). Serum bilirubin level had no significant impact on fistula development in them [18]. The pancreatic fistula was also associated with a significantly lower creatinine clearance: 59 ± 18 mL/min versus 71 ± 14 mL/min(*P* = 0.005 [18]). The impaired creatinine clearance defined as <50 mL/min precipitates acute renal failure, intra-abdominal bleeding, and sepsis, processes that predispose patients to pancreatic fistula particularly in those with obstructive jaundice [18]. Interestingly diabetes mellitus and neoadjuvant chemoradiation therapy have been shown to offer a protective benefit against pancreatic fistula, with the latter presumably causing a decrease in pancreatic exocrine secretion [19, 20] 

## 6. Operative Risk Factors

In the past two decades, various technical aspects have been scrutinized to identify operative factors associated with increased fistula rates [1–10]. Various techniques for managing the pancreatic remnant have been compared including pancreaticojejunostomy versus pancreaticogastrostomy, the duct mucosa versus invagination pancreaticojejunal anastomosis, stent versus no stent across the pancreaticoenteric anastomosis, single versus double Roux-en-Y loop reconstruction, and the use of somatostatin analogues and/or fibrin sealants [1–10].

There are other factors apart from technical consideration, of which increased intraoperative blood loss is an important risk factor for developing PF. In one of the studies pancreatic fistula group suffered significantly greater blood loss, rather than their no fistula counterparts: 1584 ± 862 mL versus 794 ± 387 mL (*P* = 0.0005) [18]. The investigators proposed that blood loss exceeding 1,500 mL is at higher risk of fistula development. The increased blood loss is likely to be associated with other factors including more advanced stages of disease (i.e., portal or superior mesenteric vein invasion, patient obesity, jaundice-associated coagulopathy and concurrent pancreatitis [5, 6, 18]). 

The risk factors of pancreatic fistula were reported following the analysis of the outcome in 233 consecutive PD [18]. They were summarized as small pancreatic ducts less than or equal to 3 mm in diameter; soft pancreatic parenchyma; ampullary, duodenal, cystic, or islet cell pathology; and intraoperative blood loss greater than 1000 mL; all of them were associated with an increased risk of developing a clinically relevant pancreatic fistulae (grades B and C) [18]. No verifiable risk factors for biochemical grade A fistulae were found in this study [18]. PF leads to delay in surgical recovery, and prolongs hospital stay and the related increase in substantial increase in hospital costs [3–8, 18, 21].

 Risk factors for pancreatic fistula following distal pancreatectomy are poorly understood. Factors that have been implicated include body mass index greater than 25 kg/m^2^, transections at the pancreatic body and absence of pancreatic duct ligation [22, 23]. In a study of 64 patients with distal pancreatectomy, 29% developed grade A fistula, 64% developed grade B, and 7% developed grade C, PF [24]. The risk factors associated with significantly higher rate of PF in this series were reported to be soft pancreatic tissue, spleen preserving procedures, and the nonuse of postoperative prophylactic octreotide [24]. Age, gender, and technique of pancreatic stump closure in this analysis were not associated with fistula development [24].

## 7. Clinical Course

The consequence of PF is increased risk of morbidity, mortality, and longer hospital stay and cost. Among the number of series recently published, the reported incidence of PF following pancreaticoduodenectomy ranged from 6% to 14% and the reported mortality from 1.4% to 3.7% [1–13]. In addition, PF is associated with other nonfistulous complications, particularly delayed gastric emptying, ileus, wound infection, intra-abdominal abscess, pancreatitis, haemorrhage, and sepsis. The hospital costs and rate of reoperation and hospital readmission are significantly increased [3–8].

The highest rate of PF however follows central pancreatectomy, which ranges from 20% to 63% among specialized center [25], in contrast to 5% following distal pancreatectomy [26]. The higher rates of PF following central pancreatectomy are presumed to be due to the creation of two pancreatic remnants in this procedure and thus two potential sites for fistula formation [25]. A study comparing the clinical and economic effects of pancreatic fistulae among patients undergoing pancreaticoduodenectomy revealed that the incidence of clinically relevant fistulae (grades B and C, according to ISGPF grading system) was 16% for pancreaticoduodenectomy, 13% for distal pancreatectomy, and 83% for central pancreatectomy [27]. Moreover it also revealed the impact of increasing risk factors on the risk of developing PF. The rate of PF in this study was 2%, 8%, 16%, 31%, and 100% in patients with number of risk factors, 0, 1, 2, 3, and 4 respectively [27]. The clinical course of these fistulas depended on the type of resection performed. PF following pancreaticoduodenectomy and central pancreatectomy led to acute manifestation and often required aggressive management in intensive care setting [2–6]. Surgical exploration when indicated was urgent and usually occurred early in the postoperative period. On the contrary patients after distal pancreatectomy seldom required aggressive management approaches nor experienced prolonged hospital stay. Unlike patients with leak post pancreaticoduodenectomy, these patients were typically discharged home rather than to rehabilitation facilities [27]. Prolonged drainage of intra-abdominal collections of more than 3 weeks and multiple hospital readmissions (usually for image-guided percutaneous) are more likely following leaks related after PD [1–8]. In a critical review of 232 cases of distal pancreatectomy, the pancreatic fistula was reported in 31% of patients (grade A = 18%, grade B = 6%, and grade C = 8%). The predominant factors associated with the leak were increased weight, higher American Society of Anesthesiologists score, blood loss greater than 1 L, increased operation time, decreased albumin level, and sutured closure of the stump without the main duct ligation. A DP with splenectomy was associated with a higher incidence of grade B or C PF. Importantly ninety-two percent of PF was successfully managed nonoperatively [28].

## 8. Preventive Approaches

### 8.1. Technical Intervention

To prevent complications following PD, various techniques of managing the pancreatic remnant have been proposed. These range from pancreatic ductal occlusion to pancreaticoenterostomy with jejunum or stomach [1–12].

### 8.2. Pancreatic Duct Occlusion

In an attempt to obviate a pancreatiocenteric anastomosis, pancreatic ductal occlusion was studied [28, 29]. Occlusion of the pancreatic duct can be achieved by simple suture ligation of the duct or injection of the duct with nonreabsorbable or reabsorbable glues [3–6]. In a study comparing pancreatic ductal occlusion by primary closure of the pancreatic duct, oversewing of the pancreatic stump and external drainage with pancreaticojejunostomy, the findings noted were that the ductal occlusion group had lower morbidity (56% versus 24%), decreased mortality (11% versus 0%), and shorter hospital stay (42.2 versus 26.4 days) [29]. However the number of cases was smaller. In another study with a slightly larger group of patients, where 86 patients received chemical and suture occlusion of the pancreatic duct compared with 83 patients with pancreaticojejunostomy, there was no significant difference found in the postoperative complication, mortality, and exocrine insufficiency [30]. The pancreatic fistula rate was significantly higher in the ductal occlusion group (17% versus 5%). After 3 and 12 months, there were significantly more patients with diabetes mellitus in the ductal occlusion group [30]. So far insufficient evidence exists to show that pancreaticoenterostomy can be replaced by pancreatic ductal occlusion.

### 8.3. Pancreaticogastrostomy

Waugh and Clagett first performed pancreaticogastrostomy (PG) in clinical practice in 1946 [31]. PG has gained favour in recent years as a possible means of reducing the incidence of pancreatic fistula [31–39]. Proponents have noticed several potential advantages. These include that pancreatic enzymes are inactivated by gastric acid environment; moreover the enzymes remain in inactive form as stomach does not contain enterokinase, which is required for conversion of trypsinogen to trypsin and subsequent activation of proteolytic enzymes. A lack of enzyme activation may help prevent autodigestion of the anastomosis. Furthermore the proximity of the pancreas to the posterior stomach wall allows potentially less tension on the anastomosis. The excellent blood supply to the stomach wall is favourable to anastomotic healing and the thickness of the stomach holds sutures well. 

There are 3 RCTs comparing pancreaticogastrostomy [32, 36, 37]. They failed to show any significant difference regarding pancreatic fistula rates, postoperative complications, and mortality. Two meta-analyses that have been published recently attempted to resolve this controversy [38, 39]. In an analysis of 11 articles along with 1 RCT, 2 prospective nonrandomized trials, and 8 cohort studies, the findings suggested that PG was safer after PD, but much of the evidence came from cohort studies [38]. The study of Wente et al. analyzed 16 articles including 3 RCTs [39]. The results indicated that all cohort studies reported superiority of PG most likely influenced by publication bias [39]. In contrast, all RCTs failed to show an advantage of a particular technique suggesting that both techniques were equally good. Based on the current evidence, PG and PJ are equivalent in terms of perioperative outcome (evidence levels 1, and 2).

### 8.4. Pancreaticojejunostomy

Pancreaticojejunostomy has been the most commonly used method of pancreaticoenteric anastomosis after PD [1–12]. This method reestablishes enteric flow of pancreatic juice after pancreaticoduodenectomy by uniting the remnant pancreatic tissue with a loop of jejunum. The jejunum is a logical choice for a pancreaticoenteric anastomosis due to its generous blood supply and mobile mesentery. Yet during the past 30 years, this technique has consistently been reported to yield on an average a 10% fistula rate (range 2 to 19%) [40]. Apart from the different positions of the jejunal loop (antecolic, retrocolic, or retromesenteric) and other variations, such as isolated Roux loop PJ, the anastomosis can be performed as an end-to-end anastomosis with invagination of the pancreatic stump in the jejunum or as end to side anastomosis with or without duct to mucosa suturing [40]. In addition to these, there are some more variations of these anastomotic techniques. 

Duct to mucosa pancreaticojejunal anastomosis allows direct contact of the pancreatic duct with jejunal mucosa, preventing direct contact of the pancreatic juice with the cut end of the pancreas and thus helping healing of the mucosa and protecting the anastomosis by embedding the pancreatic remnant under jejunal serosa [3–6, 17, 41]. Therefore, duct-to-mucosa anastomosis is theoretically more rational technique to avoid pancreatic fistulae. Since it is technically difficult to perform, duct to mucosa pancreaticojejunal anastomosis was previously recommended for patients with dilated pancreatic duct, whereas in recent years this technique has been preferred regardless of the diameter of the pancreatic duct [5, 6, 41]. Reviewing various techniques in the literatures published over the last decade, Poon et al., found that the duct-to-mucosa anastomosis was safer technique than invagination anastomosis [17].

Marcus et al., found that duct-to-mucosa anastomosis was associated with a low pancreatic fistula rate in low-risk patients with a dilated pancreatic duct or a fibrotic pancreas, whereas end-to-end invagination technique was a safer in high-risk patients with small ducts or a soft friable pancreas [42]. Suzuki et al. selected various pancreaticojejunostomy techniques according to the pancreatic texture and duct size and obtained an overall pancreatic leakage rate of 8% (4/50) [43]. The patients who developed pancreatic fistulae were all with a small duct and a soft pancreas. In that series, the incidence of pancreatic leakage rate was 6.25% in patients who underwent a duct to mucosa pancreaticojejunal anastomosis compared to 19.6% in invagination group [43]. However a prospective RCT by Bassi et al. revealed no significant difference in the morbidity and PF rate between duct to mucosa anastomosis and single-layer end-to-side pancreaticojejunostomy [44].

### 8.5. Isolated Roux Loop Pancreaticojejunostomy

Separation of the pancreaticojejunal and hepatojejunal anastomosis by an isolated Roux loop reconstruction was advocated to minimize the incidence and severity of anastomotic erosion by pancreatic juice activated by bile [45, 46]. Potential disadvantages are increased operating time and the need for an additional anastomosis. Several cohort studies have reported a low pancreatic fistula rate and its related mortality [45, 46]. In the only nonrandomized study, by Kaman et al., the data failed to show any significant difference in the pancreatic fistula rates (10% versus 12) following the isolated Roux loop pancreaticojejunal reconstruction or conventional single loop pancreaticojejunal reconstruction after PD [47]. Based on the limited evidence, the use of isolated Roux loop pancreaticojejunostomy cannot prevent pancreatic fistula formation (evidence levels 3b, and 4) [6].

## 9. Other Surgical Technical Modifications/Approaches

### 9.1. Role of Magnification in Pancreatic Anastomosis

Since a duct-to-mucosa anastomosis is crucial for good outcome, a meticulous approximation assumes great importance. Operating loupes have been used by many experts to allow precise reconstruction of pancreatic anastomosis [48]. Technical errors that may occur during anastomosis include crossing of the sutures, including both sides of the pancreatic duct while passing the sutures, taking unequal or inadequate amounts of pancreatic duct and jejunal mucosa and incorrect knot placement resulting in air knots. All these events can be avoided by using magnification. Some have reported markedly reduced incidence of PF with the operating microscope compared to operating loupes [48].

### 9.2. Blood-Supply-Based Technique of PD

One of the few modifications which have demonstrated a substantial reduction in the rate of PF after PD was proposed by Strasberg et al. [49]. A concept of vascular watershed in the pancreatic neck and its role in ischaemia of the cut surface of pancreatic remnant has been proposed by them. Based on this concept, the blood supply at the cut surface of the pancreas is evaluated in the techniques and if necessary the pancreas is cut back 1.5 cm to 2 cm to improve the blood supply (*n* = 47, 38%) [49]. Thereafter the anastomosis is performed meticulously under magnification.

### 9.3. Total Pancreatectomy

The rational for total pancreatectomy is that it allows a more extensive lymphadenectomy, obviates the risk of a leak from the pancreatic anastomosis and decreases the chance of a positive resection margin. However total pancreatectomy is associated with obligatory diabetes mellitus, decreased immunity because of splenectomy, and loss of pancreatic exocrine function [50, 51]. Hence it is no surprise that most studies have reported either worse survival or no survival difference between total pancreatectomy and standard PD [50, 51]. The indication for total pancreatectomy would include serial positive resection margin obtained on frozen section, a very soft pancreas with a potentially increased risk of PF and in patients with documented family history of multicentric disease [50, 51].

### 9.4. Pancreatic Duct Stenting

One of the technical modifications in pancreaticoenteric anastomosis is the use of a transanastomotic stent for internal and external drainage of pancreatic secretion [52–57]. The potential advantages of a pancreatic stent include diversion of pancreatic secretion from the anastomosis and facilitation for more precise placement of sutures during the anastomosis, thus protecting the pancreatic duct from suture injury and reducing the risk of iatrogenic pancreatic duct occlusion. However complications such as obstruction of the stent leading to pancreatic fistula and migration of the stent are drawbacks with transanastomotic stenting. The number of studies on pancreatic stenting is limited and the results are conflicting [52–57]. 

Internal transanastomotic stenting was reported to reduce the pancreatic fistula of pancreaticojejunal anastomosis in cohort study [52]. However in the nonrandomized study by Imaizumi et al. with 168 patients, there was no significant difference in the pancreatic fistula rates between end-to-side pancreaticojejunostomy of normal soft pancreas using stented (internal or external) method versus non stented methods (5.7% versus 6.7%) [54]. The internal pancreatic stent was evaluated by Winter et al. in an RT among 234 patients [55]. The study showed that internal pancreatic duct stenting did not decrease the frequency or the severity of the postoperative fistulas. The pancreatic fistula rates in patients undergoing PD with or without an internal pancreatic stent were 11.3 and 7.6%, respectively [55].

 An external stent has a theoretical advantage of more complete diversion of pancreatic secretion away from the pancreaticojejunal anastomosis and prevents the activation of pancreatic enzyme by bile [56]. The nonrandomized study by Ohwada et al. showed equivalent outcomes for external and internal pancreatic stenting of duct-to-mucosa pancreaticojejunostomy after PD [56]. However the RCT by Poon et al. showed that among the 120 patients who were externally stented, pancreatic fistula rate was significantly lower in them compared to the nonstented group (6.7% versus 20%) [57]. A recent study of 158 patients who underwent PD and were randomized to receive an external stent or no stent revealed that the stented group had a significantly lower rate of pancreatic fistula (26% versus 42%), *P* = 0.034., morbidity (41.5% versus 61.7%), *P* = 0.01, and delayed gastric emptying (7.8% versus 27.2%) *P* = 0.001 [58]. Based on the current evidence, it is unclear whether drainage of the pancreatic duct with a stent can reduce the pancreatic fistula rate after PD (evidence levels 2 and 3 b). Hence an internal and external pancreatic duct stent may or may not be placed across these anastomosis. Though some of the evidence shows that external stenting technique is superior, it appears that as long as tension-free anastomosis between well-perfused tissues is performed, employing fine sutures and using the same technique, any type of pancreatic anastomosis should result in a good outcome.

### 9.5. Pharmacologic Intervention

Somatostatin is a potent inhibitor of endocrine and exocrine functions. The synthetic peptide octreotide contains the same amino acid sequences essential to the activity of somatostatin, while conferring resistance to enzyme degradation, resulting in a long acting stable analog suitable for subcutaneous administration. The rationale of its use following PD is that by decreasing the volume of pancreatic secretion, the pancreatic fistula rate would be decreased because of which the pancreaticoenteric anastomosis would heal better. Adverse effects of somatostatin and its analogues include nausea, flatulence, diarrhea, steatorrhea, pain at the injection site, and abdominal discomfort. There are 11 RCTs involving 2023 patients in whom the somatostatin analogue was examined [59–69]. Five RCTs from Europe [59–69] and 1 RCT from Asia [65] showed the benefit of perioperative use of somatostatin analogues to decrease the postoperative complication rate. On the other hand, 2 recent RCTs from Europe [66, 67] and 3 RCTs from USA tates failed to show benefit [11, 15, 64]. Two recent meta-analyses have been published. Connor et al. analyzed 10 studies and showed that somatostatin and its analogues reduced rate of biochemical fistula but not the incidence of clinical anastomotic disruption [68]. In another report involving seven studies, the perioperative octreotide administration was associated with significant reduction of pancreatic fistula rate after pancreatic surgery [69]. However the risk reduction was not associated with a significant difference in postoperative mortality. Conclusions drawn from these meta-analysis should be cautionary, as pooling of data from these RCTs was difficult because there was considerable heterogenicity in these studies from end point measures, definition of outcome measurements, treatment regimens, pathologic findings, type of pancreatic surgery, and anastomotic technique.

Hence the prophylactic use of perioperative somatostatin and its analogues to prevent pancreas-related complications after pancreatic surgery remains controversial. It does not result in a reduction of mortality. However the efficacy of prophylactic octreotide is reported to be improved, by selective administration in the setting of high risk glands, including patients with either soft glands or small pancreatic duct, in those harbouring ampullary, duodenal cystic or islet lesions, or in case where intraoperative blood loss is excessive [3]. Prophylactic octreotide did not influence clinically relevant fistula rates among low-risk glands [3]. There is a need for RCTs with standardization in definition of outcome measurements, treatment regimen, surgical technique, and stratification of risk factors.

## 10. Management Approaches

In recent years, pancreatic surgeons have strived hard to reduce postoperative PF by developing numerous novel strategies [1–13]. But the cornerstone of minimizing the potentially devastating effect of PF is to recognize this complication as soon as it develops and to institute appropriate treatment measures promptly [2–8]. The suspicion of PF begins whenever there is a deviation in the normal clinical course of a patient who has just undergone a major pancreatic surgery. This includes a patient who develops unexpected upper abdominal discomfort (often associated with fever), leukocytosis, increasing tachycardia, or just feels unwell after an apparently “normal” initial postoperative recovery [3, 5, 6]. This suspicion may be substantiated by the presence of high amylase content of the drain, a persistently high drain output, altered drain colour and quality, and other complications such as severe wound infection and haemorrhage. Routine radiological investigation is generally not recommended as it is not necessary [3, 6]. Once the diagnosis of PF is established, aggressive and appropriate conservative management is the key to successful outcome in majority of the patients [2–12]. However, interventional radiological assistance is sometimes required, but repeat surgery is rarely indicated [3, 7, 35].

## 11. Conservative Management

Nonoperative management of pancreatic fistula includes treatment for postoperative ileus and intra-abdominal collection and is successful in about 90% of cases [70, 71]. Clinical evaluation of the patient at short intervals is of outmost importance. Patients are kept nil per oral and provided adequate hydration. In those patients who have not yet tolerated oral or those who are presenting with complication on or after the 10th postoperative day, would require parenteral nutritional support [3–6]. Nutrition can also be maintained by enteral nutrition (through an operatively placed nasojejunal tube or feeding jejunostomy) in less severe cases. Empiric antibiotics are given if signs of infection (i.e., fever, leukocytosis, purulent discharge, erythema, warmth, tenderness) are present and adjusted depending on information from gram stains or cultures. Intra-abdominal drains are left in situ until daily drainage volumes approach 50 mL per day; patients can be discharged home as long the character of the drainage is not purulent or particulate. Cautious drain management (i.e., in situ drainage is indicated in patients with high-output drainage (greater than 200 mL per day) and amylase rich effluent (greater than 1000 IU/L) [2–6]). Therapeutic octreotide may be administered to reduce pancreatic secretions, typically until oral intake resumes and/or hospital discharge occurs. All along abdominal drains and the main wound would require close attention [3–6]. 

The interventional radiologist may play a crucial role by image-guided repositioning of operatively placed drains and insertion of percutaneous catheters to drain collections seen in CT scan [71, 72]. Delayed haemorrhage following PF is a major concern and is probably best managed by angiography and embolization of the bleeding vessel [73, 74]. This treatment is successful in stopping the bleeding in 80% of patients [73, 74]. The prognosis of patients with postpancreatectomy haemorrhage depends on whether or not PF is present. The decision-making should be guided by factors such as the time of onset of the bleeding, presence of PF, vascular pathology, and the underlying disease process [73, 74]. The failure to successfully control haemorrhage by conservative measures like angiographic embolization may necessitate repeat surgery [73, 74].

**Figure 1:**
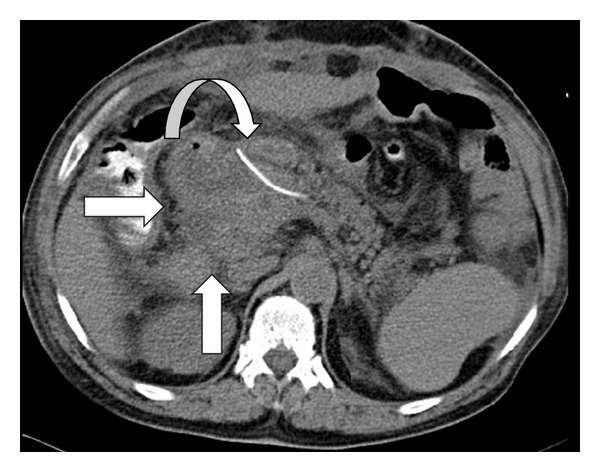
CT scan carried out on the 9th postoperative day following pancreaticoduodenectomy reveal peripancreaticojejunal anastomosis collection (straight arrows). This grade B pancreatic fistula was successfully managed by CT guided aspiration. The internal pancreatic duct stent is also seen (curved arrows).

Intra-abdominal collection can be dealt by CT or ultrasound-guided percutaneous drainage and is considered at the discretion of the surgeon (Figure 1). Some employ this modality in the presence of large fluid collections that have not responded to conservative therapies, but which are amenable to drainage [2–6, 13]. Surgical exploration is seldom required but is indicated when anastomotic dehiscence is suspected and for patients who deteriorate clinically often in the setting of a non drainable abscess, sepsis, or multiple-organ dysfunction. The options that are considered include wide peripancreatic drainage of an abscess or fluid collection, revision of the initial pancreaticoenteric anastomosis, conversion to an alternative pancreaticoenteric anastomosis, or completion pancreatectomy (i.e., total pancreatectomy). 

Surgical peripancreatic drainage, which is proposed as a safer alternative, to completion pancreatectomy, may be suitable for less severe postoperative pancreatic fistula. However in patients with severe postoperative pancreatic fistula with disruption of the pancreaticojejunostomy, simple peripancreatic drainage might not be effective [14, 75]. Completion pancreatectomy, which is often used as a salvage procedure in such instance, however is associated with high perioperative mortality ranging from 75% to 100% with severe morbidity of brittle diabetes [14, 75, 76]. The associated morbidity of type 1 diabetes and exocrine insufficiency constitute lifelong morbidity requiring frequent hospitalization. Hence to avoid these complications, some have recommended salvage pancreaticogastrostomy [75]. The relatively favourable outcome following this procedure is attributable to the anatomic position of the pancreas, which makes PG relatively easy even in the presence of pancreatitis or peritonitis, in addition to failure of activation of enzyme in the absence of enterokinase in the stomach and feasibility of drainage pancreatic juice by continuous gastric aspiration [75].

## 12. Conclusions

Pancreatic resection is now considered a safe procedure, when performed in high-volume centers. It is associated with low mortality rates, shorter hospital stays, and modest improvements in postoperative morbidity. PF from pancreaticoenteric anastomosis, however, continues to be a significant problem after PD.

The definition and grading system put forth by the international study group on PF is now standard consensus definition for PF and helps in comparing results of various reports. Among the numerous risk factors for the clinically relevant fistula, the most likely are soft pancreatic parenchyma, small pancreatic ducts, resection for ampullary, duodenal, cystic and islet cell pathology, and excessive blood loss. Preventive approaches to reduce the rate of PF include technical and anastomotic modifications, particularly the employment of duct-to-mucosa anastomosis. Use of prophylactic octreotide is found to be beneficial in select group of patients, like those at high risk for developing PF. Successful management of this serious complication depends on early detection, which requires a high index of clinical suspicion. Analysis of drainage fluid is the principal diagnostic tool, but computed tomography, ultrasound, and pancreaticography provide additional information. Nonoperative management strategies form the cornerstone of management in majority of the patients and include managing fluid balance, providing parenteral nutritional support and administrating antibiotics or octreotide. Image-guided drainage or surgical exploration is indicated if large fluid collections persist and/or patients deteriorate clinically. The indication for surgical intervention in PF includes worsening clinical parameters, signs of spreading peritonitis, severe wound infection, wound dehiscence, and disruption of pancreatic anastomosis. The surgical intervention may be of minimal nature by draining the peripancreatic fluid or involve salvage completion pancreatectomy. 

In spite of long standing experience with PF, pancreatic surgeons continue to invest significant effort to improve perioperative management of this complication. As current fistula rates hover around 15%, the future success of pancreatic surgery will require better diagnostic approaches, novel management algorithms, randomized clinical trials, multi-institutional investigations, and multidisciplinary collaboration in order to reach negligible level of PF.

## Figures and Tables

**Table 1:** The different components of previously used definitions of pancreatic fistula prior to the new grading System by the international study group for pancreatic fistula (ISGPF) (presented in Table 2).

(i) Output >10 mL/day of amylase rich fluid on postoperative day 5 or for >5 days	
(ii) Output >10 mL/day of amylase rich fluid on postoperative day 8 or for 8 days	
(iii) Output of >50 mL/day of amylase rich fluid after postoperative day 11 or for more than 11 days	

**Table 2:** Criteria for grading pancreatic fistula (ISGPF classification scheme). Signs of infection include elevated body temperature >38°C, leukocytosis and localized erythema, induration, or purulent discharge. Readmission is any hospital admission within 30 days following hospital discharge from the initial operation. Sepsis is the presence of localized infection and positive culture with evidence of bacteraemia (i.e., chills, rigors, elevated WBC) requiring IV antibiotic treatment, or hemodynamic compromise as demonstrated by high cardiac output and low SVR within 24 h of body temperature >38°C.

Criteria	No fistula	Grade A fistula	Grade B fistula	Grade C fistula
Drain Amylase level	<3 times normal serum amylase	>3 times normal serum amylase	>3 times normal serum amylase	>3 times normal serum amylase
Clinical conditions	Well	Well	Often well	Ill appearing
Specific treatment	No	No	Yes/no	Yes
US/CT if obtained	Negative	Negative	Negative/positive	positive
Persistent drainage (>3 weeks)	No	No	Usually yes	Yes
Signs of infection	No	No	Yes	Yes
Readmission	No	No	Yes/no	Yes/no
Sepsis	No	No	No	Yes
Reoperation	No	No	No	Yes
Death related to fistula	no	no	no	Yes

Adapted from [8].
